# Acute Renal Artery Thrombosis Following Delayed Postoperative Anticoagulation in Atrial Fibrillation: A Case Report Supporting Apixaban Monotherapy

**DOI:** 10.7759/cureus.93720

**Published:** 2025-10-02

**Authors:** Muhammad Rafeeq Kureemun, Muhammad Shariq Rahemtoola, Anas Abdallah Ibrahim Husein, Sanjay Dalmia

**Affiliations:** 1 Internal Medicine, North Manchester General Hospital, Manchester, GBR; 2 Urology, North Manchester General Hospital, Manchester, GBR; 3 General Surgery, North Manchester General Hospital, Manchester, GBR

**Keywords:** acute kidney injury, apixaban, atrial fib, direct oral anticoagulants (doac), post-mastectomy complications, postop complication, renal artery thrombosis, renal infarction, secondary hypertension

## Abstract

Renal artery thrombosis is a rare but potentially devastating vascular emergency, often misdiagnosed due to its nonspecific symptoms. Atrial fibrillation (AF) is a well-known cause of systemic thromboembolism, yet embolisation to the renal arteries remains uncommon. Timely recognition and appropriate anticoagulation are essential to minimise irreversible renal injury.

We report the case of an 84-year-old woman with a history of AF (CHA2DS2-VASc score of 5) who developed acute unilateral renal artery thrombosis shortly after undergoing a unilateral mastectomy with sentinel lymph node biopsy. Anticoagulation with apixaban had been appropriately held preoperatively due to bleeding risk, but its resumption was delayed until five days postoperatively. The patient presented with acute flank pain, hypertension, and an acute kidney injury on postoperative day five. A CT abdomen pelvis confirmed a left renal artery thrombosis. Remarkably, a CT angiogram of the whole aorta, performed within 24 hours of restarting apixaban, showed resolution of the thrombus, although renal function did not improve by discharge.

This case contributes to the limited but growing body of literature suggesting that direct oral anticoagulant (DOAC) monotherapy, specifically apixaban, is effective in managing renal artery thrombosis. It also reinforces the need for clinicians to consider the timely resumption of anticoagulation and to carefully reassess perioperative management in high-risk AF patients to prevent irreversible thromboembolic complications.

## Introduction

Acute renal artery thrombosis is a rare but serious vascular emergency that is often underdiagnosed due to its nonspecific clinical presentation. With an estimated incidence of less than 1% among patients presenting with acute flank pain, it is frequently mistaken for more common conditions such as renal colic, pyelonephritis, or musculoskeletal pain [1]. The underlying pathophysiology typically involves either in situ thrombosis or embolisation, resulting in abrupt cessation of renal perfusion and subsequent infarction [2].

Atrial fibrillation (AF), the most common sustained cardiac arrhythmia, is a well-established risk factor for thromboembolic events. Risk stratification is commonly performed using the CHA2DS2-VASc score, which incorporates age, comorbidities, and sex to estimate stroke risk [3]. While clinical focus typically centres on stroke prevention, systemic embolisation to organs such as kidneys, spleen and mesentery is also recognised, particularly in patients with elevated CHA2DS2-VASc scores who are not adequately anticoagulated [4].

Direct oral anticoagulants (DOACs) such as apixaban and rivaroxaban have become standard therapy for stroke prevention in AF, and there are increasing reports of their successful use in treating visceral arterial thrombosis, including renal infarction. Their rapid onset of action and predictable pharmacokinetics make them an attractive option compared with traditional vitamin K antagonists or heparin bridging, though their efficacy in this setting still needs to be ascertained [5]. In the perioperative setting, anticoagulation must be carefully managed by balancing the individual’s thromboembolic risk against the procedure’s bleeding risk. Timely resumption postoperatively is critical to minimise the risk of systemic embolisation [6].

We present a case of acute unilateral renal artery thrombosis in a patient with known AF who had recently undergone breast surgery. The thrombus resolved within 24 hours of reinitiating apixaban, as confirmed by a CT angiography of the whole aorta. Although anticoagulation had been appropriately withheld due to the procedural bleeding risk, it was not resumed until five days postoperatively. This case highlights the complexities of perioperative anticoagulation in high-risk patients and adds to the emerging evidence supporting the efficacy of DOAC monotherapy, specifically apixaban, in managing renal artery thrombosis.

## Case presentation

We present the case of an 84-year-old female patient who attended the accident and emergency department (A&E) with a two-day history of left-sided abdominal pain and two episodes of non-bilious vomiting. She denied any associated symptoms such as palpitations, fever, urinary symptoms, or recent trauma. Notably, she had not opened her bowels for five days, although she was still passing flatus. She had been treated in the community for presumed constipation with an enema the day prior to admission.

Her past medical history included AF, for which she was on apixaban 5 mg twice daily. However, anticoagulation had been withheld for a total of eight days: two days prior to surgery, on the day of surgery, and for five days postoperatively. She had undergone a left simple mastectomy with sentinel lymph node biopsy five days before her presentation, following a diagnosis of symptomatic left breast cancer. On the day of admission, she was scheduled to restart apixaban. Her other comorbidities included diet-controlled type 2 diabetes mellitus, hyperlipidaemia managed with atorvastatin 20 mg at night, and hypertension managed with lercanidipine 10 mg once daily. She was also on furosemide 40 mg twice daily. No changes were made to the above three medications perioperatively that would significantly alter thromboembolic or bleeding risk. Postoperatively, she had been using codeine phosphate 30 mg four times daily as needed for postoperative pain, which likewise had no impact on thromboembolic or bleeding risk.

Upon arrival, she was haemodynamically stable with a temperature of 36.5°C, blood pressure of 185/83 mmHg, and a heart rate of 91 beats per minute, which was irregularly irregular, consistent with her known AF. Her respiratory rate was 18 breaths per minute, and she maintained oxygen saturation of 99% on room air. On examination, she was noted to have generalised abdominal tenderness, with maximal discomfort in the left iliac fossa. Bowel sounds were reduced; a digital rectal examination revealed an empty rectum.

Initial blood investigations demonstrated a leukocytosis of 11.40 x 10^9^/L (reference range: 4.00-11.00 x 10^9^/L), with a neutrophilia of 9.17 x 10^9^/L (reference range: 1.80-7.50 x 10^9^/L) and a mildly elevated monocyte count of 1.09 x 10^9^/L (reference range: 0.20-1.00 x 10^9^/L). C-reactive protein (CRP) was elevated at 27.00 mg/L (reference range: 0.00-5.00 mg/L), and alanine transaminase (ALT) was raised at 136.00 IU/L (reference range: 1.00-36.00 IU/L). There was evidence of stage 1 acute kidney injury, with a creatinine level of 115.00 µmol/L, previously 71.00 µmol/L (reference range: 45.00-84.00 µmol/L), and an estimated glomerular filtration rate (eGFR) of 38.00 ml/min/1.73 m², down from 68.00 ml/min/1.73 m². This was compared to the blood results 11 days prior during the preoperative assessment (Table 1). A venous blood gas showed a normal pH and a lactate of 1.20 mmol/L, within the normal reference range. Neither a urinalysis nor a lactate dehydrogenase (LDH) level, commonly recommended to support the diagnosis of renal infarction [1], was obtained, which limited the ability to distinguish renal artery thrombosis from close mimics such as renal colic, a passed kidney stone, pyelonephritis, or gastroenteritis.

**Table 1:** Blood results on the preoperative assessment day, on admission, and on discharge This table compares the laboratory blood values obtained during the preoperative assessment (11 days prior to admission), on hospital admission, and on the day of discharge (four days post admission). Reference ranges are provided in the second column. The test results for sodium and alkaline phosphatase (ALP) have been omitted due to their non-clinical relevance to the case. N/A: test not performed

Test	Reference range & units	Preoperative assessment bloods – 11 days pre-admission	Admission bloods	Bloods on day of discharge – 4 days post-admission
White blood cells (WBC)	4.00-11.00 x 10^9 ^/L	7.20	11.40	9.70
Neutrophils	1.80-7.50 x 10^9 ^/L	3.82	9.17	4.51
Monocytes	0.20-1.00 x 10^9 ^/L	0.69	1.09	1.11
Potassium	3.50-5.30 mmol/L	3.90	3.60	4.50
Urea	2.50-7.80 mmol/L	5.60	6.30	6.40
Creatinine	45.00-84.00 µmol/L	71.00	115.00	118.00
eGFR	>90.00 ml/min/1.73m^2^	68.00	38.00	37.00
Alanine transaminase (ALT)	1.00 -35.00 IU/L	36.00	136.00	N/A
C-reactive protein (CRP)	0.00-5.00 mg/L	N/A	27.00	N/A

**Figure 1:**
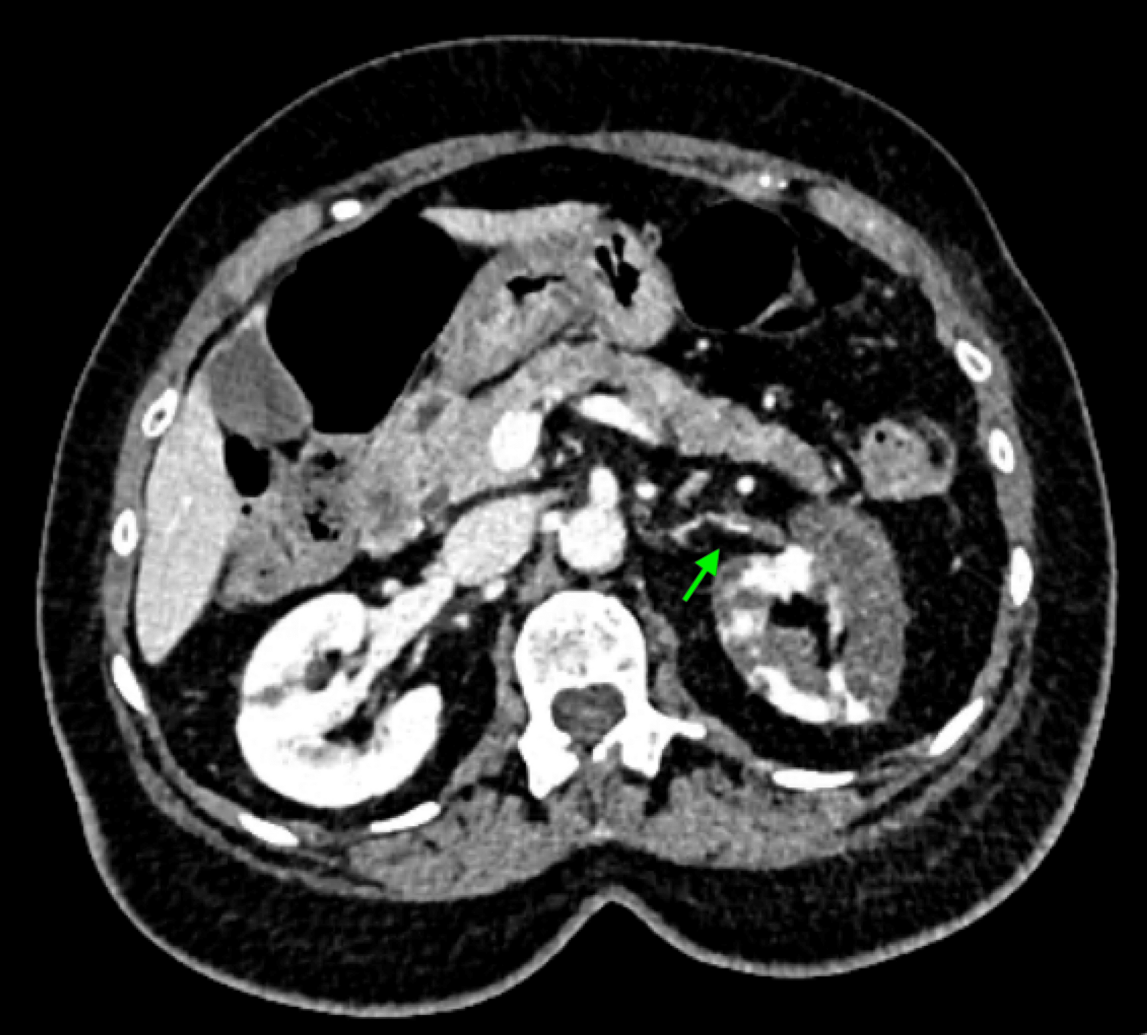
Axial contrast-enhanced CT abdomen and pelvis (CTAP) The green arrow highlights a filling defect in the left renal artery, consistent with left renal artery thrombosis. There is reduced enhancement of the left kidney compared to the right, suggesting impaired perfusion.

Given her abdominal symptoms of vomiting, abdominal pain, and not opening bowels alongside her examination findings of reduced bowel sounds, the clinical impression was that of possible bowel obstruction. She was therefore commenced on intravenous rehydration with Hartmann’s solution (1000 mL over six hours), and a CT of the abdomen and pelvis with contrast (CTAP) was performed to evaluate for obstruction or perforation. Interestingly, while the CTAP did not show evidence of bowel obstruction or perforation, it revealed an incidental finding of thrombus within the left renal artery, involving both the origin and distal branches, as well as segmental arteries supplying the lower pole of the kidney (Figure 1). This was associated with multifocal infarction of the left kidney, particularly affecting the lower pole, with sparing of parts of the upper and mid-pole cortex.

Following a multidisciplinary discussion with the vascular surgery team, it was agreed that the patient should be managed conservatively, given the absence of haemodynamic instability and the resolving nature of her abdominal symptoms. Apixaban was promptly restarted at 5 mg twice daily, and additional investigations were arranged to explore the source and extent of embolic disease.

**Figure 2:**
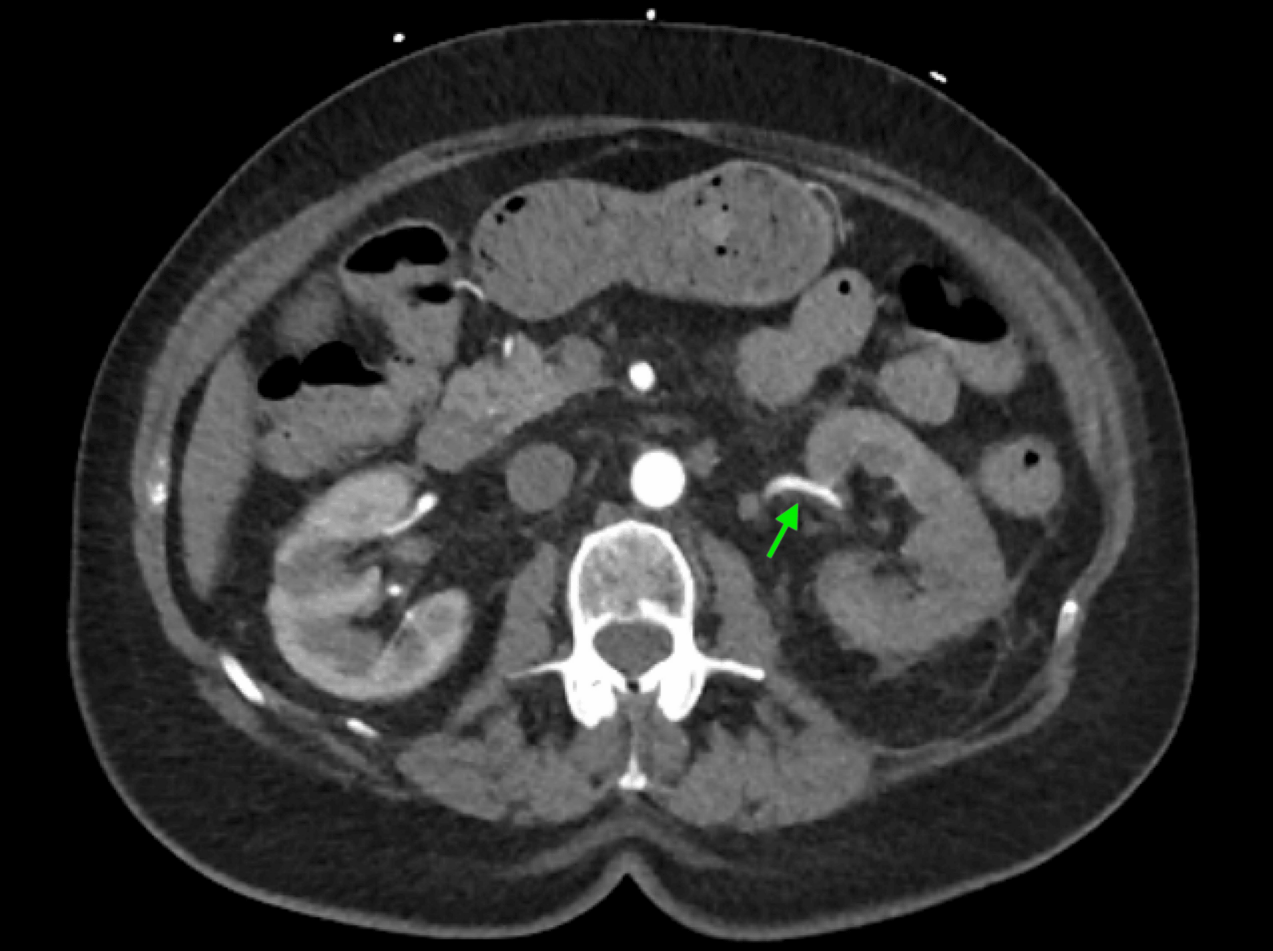
Axial CT angiogram of the whole aorta The green arrow points to the left renal artery, which now shows normal contrast opacification, indicating resolution of a previously present thrombus.

A CT angiogram of the whole aorta, performed the following day after the patient had received two doses of apixaban, demonstrated patency of both renal arteries (Figure 2). There was a moderate calcific stenosis at the origin of the left renal artery, but notably, the previously seen thrombus had resolved, suggesting an early reperfusion with anticoagulation. A 24-hour Holter monitor confirmed persistent AF, with a mean heart rate of 86 beats per minute (bpm) (range: 72-128 bpm) and 67 premature ventricular contractions (PVCs). Transthoracic echocardiography revealed severe tricuspid regurgitation and mild mitral regurgitation, but no evidence of intracardiac thrombus, vegetations, or other structural abnormalities. The left ventricular systolic function appeared preserved, though it was not formally quantified.

During her hospital stay, the patient's abdominal symptoms improved with continued IV hydration, anticoagulation with apixaban and a second enema to address her constipation. Her renal function remained static, with a creatinine of 118.00 µmol/L and an eGFR of 37 ml/min/1.73 m² over the following four days. This was considered to reflect her new baseline renal function, likely due to partial infarction. Her medications were adjusted to account for the reduced renal clearance, and she was subsequently discharged home with arrangements for renal and cardiology outpatient follow-up. Long-term renal function data were unavailable, limiting outcome assessment. 

## Discussion

We present a case of postoperative acute renal infarction secondary to thromboembolism in the setting of AF, following a total of eight-day interruption of anticoagulation: two days prior to surgery, on the day of surgery, and five days postoperatively. The patient presented with generalised abdominal pain, most marked in the left iliac fossa, alongside elevated blood pressure and biochemical evidence of acute kidney injury. A CT abdomen and pelvis revealed acute thrombosis of the left renal artery with multifocal infarction involving the lower pole of the left kidney.

Following the reinitiation of anticoagulation with apixaban 5 mg twice daily, a CT angiogram of the whole aorta performed after two doses of the anticoagulant showed resolution of the previously observed thrombus. However, her renal function remained impaired and did not return to baseline by the time of discharge, four days later.

Our case raised two important clinical questions. Firstly, what is the optimal timing of restarting apixaban in a patient with AF following surgery (specifically a unilateral mastectomy with sentinel lymph node biopsy), balancing the risk of thromboembolism and postoperative bleeding? Secondly, are renal artery emboli secondary to AF particularly responsive to DOAC therapy, especially apixaban, given the resolution of the left renal artery thrombus on the CT angiogram imaging after only two doses of apixaban?

Previous clinical case reports [2] have already highlighted the challenge of diagnosing renal artery thrombosis due to its non-specific clinical presentation, as well as the different imaging modalities required for accurate identification [7]. Our discussion will centre on the two aforementioned clinical questions, which are more pertinent to our case.

To address the first one, we have to gain an understanding of the current guidelines on perioperative DOAC management based on the recent evidence.

Guidelines on perioperative DOAC management

The perioperative management of DOACs is guided by a combination of factors, including the specific pharmacokinetics of the drug (particularly its half-life), the patient’s renal function and thromboembolic risk, and the bleeding risk associated with the planned surgical procedure. Several professional societies [8] have issued guidelines based on the accumulating evidence from key clinical trials.

To consolidate the evolving body of evidence, the 2021 European Heart Rhythm Association (EHRA) Practical Guide [8] incorporated data from landmark studies such as Perioperative Anticoagulant Use for Surgery Evaluation (PAUSE) [9] and Perioperative Factor Xa Inhibitor Discontinuation in Patients With Atrial Fibrillation Undergoing Minimal to Low Bleed Risk Procedures (PERIXa) [10], providing a comprehensive framework for the perioperative management of DOACs in patients with AF. Table 2 summarises the recommendations of both the PAUSE study protocol and the EHRA 2021 guidance.

**Table 2:** Summary of perioperative DOAC (apixaban) management: PAUSE study and EHRA 2021 guidelines *Both the PAUSE study and the EHRA guidelines provide a list of procedures which are classified as low or high bleeding risk. **Please note that the interruption duration may require adaptation based on the individual patient characteristics (including age, stroke risk, history of bleeding complications, concomitant medication, kidney function, etc.). ***Apixaban resumption is dependent upon whether haemostasis has been achieved. CrCl: creatinine clearance (mL/min); DOAC: direct oral anticoagulant; PAUSE: Perioperative Anticoagulant Use for Surgery Evaluation; EHRA: European Heart Rhythm Association

Source	Procedure bleeding risk*	Apixaban interruption duration**	Apixaban resumption postoperatively***
1. PAUSE study protocol [9]	Low	24 hours prior	24 hours
	High	48 hours prior	48–72 hours
2. EHRA 2021 guidance [8]	Low	24 hours prior (if CrCl≥30 mL/min), 36 hrs prior (if 15≤CrCl<30 mL/min)	24 hours
	High	48 hours prior (if CrCl≥15 mL/min)	48–72 hours

Of note, in the per-protocol analysis of the apixaban cohort in the PAUSE study [9] (where the participants adhered completely to the above protocol), the 30-day postoperative rate of major bleeding was 1.20% (with a 95% confidence interval ranging from 0.00% to 1.89%), and the rate of arterial thromboembolism was 0.19% (with a 95% confidence interval ranging from 0.00% to 0.56%). These low rates underscore the safety of this standardised strategy, without the need for heparin bridging or coagulation testing.

The EHRA guide [8] also emphasises that, in certain surgical procedures, resuming full-dose anticoagulation within the first 48-72 hours postoperatively may pose a bleeding risk that exceeds the thromboembolic risk associated with AF. In such cases, thromboprophylaxis with a prophylactic dose of low molecular weight heparin (LMWH) can be initiated six to eight hours after surgery, with therapeutic dose DOACs delayed by ≥ 48-72 hours, provided haemostasis is secured. This will provide some degree of thrombotic protection while minimising bleeding risk.

Both interruption and resumption of DOAC therapy should be tailored to individual patient factors, including renal function, prior bleeding history and thromboembolic risk.

Risk stratification in our patient

Bleeding Risk

The timing of postoperative DOAC resumption is closely guided by the bleeding risk associated with the procedure. Although unilateral mastectomy with sentinel lymph node biopsy is not explicitly classified, the perioperative management in our case, specifically withholding anticoagulation for two days preoperatively, suggests the procedure was treated as a high bleeding risk. Full-dose anticoagulation was resumed on postoperative day five.

Thromboembolic (Arterial) Risk

In our patient, a CHA2DS2-VASc score of five (calculated based on the blood results and observations during the preoperative assessment) indicates a high (arterial) thromboembolic risk, corresponding to a 6.7% annual risk of systemic embolism [3]. Moreover, given that she has underlying breast cancer, it puts her in a pro-thrombotic state.

Venous Thromboembolism (VTE) Risk

Her VTE risk was assessed using a modified Caprini score, taking into account the following factors: age above 70 years, BMI between 25 and 29 kg/m², use of a self-aid device for mobility, operative time under 90 minutes, and presence of an active malignancy (invasive breast cancer). Based on these criteria, she was classified as having moderate VTE risk, for which the recommended prophylactic measures included early postoperative mobilisation, adequate hydration, intraoperative intermittent pneumatic compression (flowtrons), and the use of thrombo-embolic deterrent (TED) stockings. Routine postoperative prophylactic LMWH was not indicated.

Therefore, our patient had a high thromboembolic risk (associated with AF) and a high bleeding risk (associated with the type of surgery).

Postoperative management gaps

According to the patient’s records, the decision to resume apixaban on postoperative day five was documented in the operative note. As discussed earlier, the timing of DOAC resumption should be guided by the confirmation of adequate haemostasis. Notably, both the operative note and nursing documentation (recorded two hours post surgery) stated that haemostasis had been achieved and there was no active bleeding. Of note, a further postoperative reassessment by the surgical team may have supported an earlier resumption of apixaban, potentially reducing the duration the patient remained without anticoagulation.

As summarised in Table 2, DOACs should typically be restarted within two to three days postoperatively in high bleeding risk patients, once haemostasis is secured. In our case, apixaban was restarted on day five postoperatively, resulting in a total of eight days off anticoagulation (two days preoperatively, on the day of surgery, and five days postoperatively). This is clinically relevant in light of the Periprocedural DOAC Management study [11], which found that patients off anticoagulation for more than six days had 90% higher odds of thrombotic events compared to those with ≤ six days off (95% confidence interval: 1.43-2.5), indicating a statistically significant association. However, one important limitation of this study is its observational nature without a standardised protocol. Thus, while the association is statistically significant, causality cannot be confirmed without randomised data or stricter adjustment for these variables.

In addition, given that both the bleeding risk and thromboembolic risk were significant, it could be argued that prophylactic low-dose LMWH should have been initiated postoperatively once haemostasis was established (supported by the 2021 EHRA guide [8]). No patient-specific factors were identified that could justify withholding LMWH: haemostasis had been achieved, there were no bleeding-related concerns, no comorbidities that increased haemorrhagic risk, and no medications or absorption issues that would contraindicate LMWH use. While this does not offer full protection against AF-related embolism, it provides partial anticoagulant coverage instead of leaving the patient entirely unprotected. This could serve as a bridging strategy, with plans to escalate to a therapeutic dose of LMWH or resume DOAC therapy once the bleeding risk has sufficiently decreased. The rationale is to mitigate thromboembolic risk without significantly increasing bleeding complications.

This case highlights the clinical challenge of navigating bleeding versus thromboembolic risk in the perioperative setting, particularly in high-risk AF patients, and underscores the importance of individualised reassessment to guide timely anticoagulation decisions.

Another notable aspect of our case is the rapid resolution of a left renal artery thrombus following just two doses of apixaban, as demonstrated on CT angiography of the whole aorta. This raises an important clinical question: are renal artery emboli secondary to AF particularly responsive to DOAC therapy?

Role of DOACs in the management of renal artery thrombosis

Evidence guiding the management of renal artery thrombosis remains limited, with most data stemming from case reports and small case series [12]. The primary goals of treatment are to alleviate symptoms, preserve renal function and optimise blood pressure control. Therapeutic strategies include anticoagulation, systemic or catheter-directed thrombolysis, surgical thrombectomy, aortorenal bypass, and endovascular techniques such as thrombectomy, angioplasty and stenting.

The optimal treatment modality for renal artery embolism remains controversial. No randomised clinical trials have established the superiority of one approach over another. While anticoagulation is a common starting point, some clinicians favour endovascular interventions such as thrombolysis or thrombectomy, particularly where there is potential for renal salvage. However, these procedures require experienced interventional radiologists and carry a risk of procedural complications.

Although the literature remains sparse, there are emerging reports of DOAC monotherapy for renal artery thrombosis. Costa et al. (2024) [13] described a patient with bilateral renal artery thrombosis treated with apixaban monotherapy over nine months, resulting in gradual renal function improvement and dialysis withdrawal. In their literature review, most previously reported cases used warfarin, with only one involving paroxysmal AF. Moreover, Al-Sadi et al. (2022) [14] reported a case of unilateral renal artery thrombosis due to AF that was successfully treated with dabigatran, showing preserved renal artery flow on CT scan four months after treatment.

It is also important to distinguish radiographic resolution of thrombus from functional renal recovery. Restoration of blood flow does not guarantee improved kidney function, particularly if infarction has already occurred. In our case, although the thrombus resolved rapidly, renal function did not improve, suggesting that irreversible damage may have already taken place. Nonetheless, early recognition and treatment are associated with better outcomes and may limit the extent of ischaemic injury.

## Conclusions

Our case adds to the limited but growing body of evidence supporting the potential efficacy of DOAC monotherapy, specifically apixaban, in achieving rapid resolution of renal artery thrombosis, as demonstrated by radiographic clearance within 24 hours of DOAC initiation. However, renal function did not improve, underscoring the importance of early diagnosis before irreversible infarction occurs. This observation supports that apixaban may be effective in selected cases, but it should be interpreted with caution, given the limited evidence currently available. Further clinical studies and detailed case reporting are needed to define the role of DOACs in the conservative management of renal artery thrombosis.

Similarly, this case also reinforces the need for individualised dynamic reassessment of perioperative anticoagulation in AF patients. The delayed resumption of apixaban combined with the non-consideration of low-dose LMWH and a lack of a further surgical review to confirm haemostasis likely contributed to the development of a renal artery thromboembolism, emphasising the need for individualised anticoagulation planning.
